# Cognitive Impairment in Genetic Parkinson's Disease

**DOI:** 10.1155/2021/8610285

**Published:** 2021-12-30

**Authors:** A. Planas-Ballvé, D. Vilas

**Affiliations:** ^1^Neurology Department, Hospital Sant Joan Despí Moisès Broggi, Consorci Sanitari Integral, Sant Joan Despí, Spain; ^2^Neurology Department, Hospital Universitari Germans Trias i Pujol, Badalona, Barcelona, Spain

## Abstract

Cognitive impairment is common in idiopathic Parkinson's disease (PD). Knowledge of the contribution of genetics to cognition in PD is increasing in the last decades. Monogenic forms of genetic PD show distinct cognitive profiles and rate of cognitive decline progression. Cognitive impairment is higher in *GBA-* and *SNCA*-associated PD, lower in *Parkin-* and *PINK1*-PD, and possibly milder in *LRRK*2-PD. In this review, we summarize data regarding cognitive function on clinical studies, neuroimaging, and biological markers of cognitive decline in autosomal dominant PD linked to mutations in *LRRK2* and *SNCA*, autosomal recessive PD linked to *Parkin* and *PINK1,* and also PD linked to *GBA* mutations.

## 1. Introduction

Cognitive impairment is common in Parkinson's disease (PD). Approximately 20–33% of patients have mild cognitive impairment (MCI) at the time of diagnosis [1, 2], and up to 80% of patients develop dementia during the course of the disease [3, 4]. Some factors clearly related to cognitive impairment in PD are older age and longer disease duration [5, 6]. Cognitive domains that are usually impaired are attention and visuospatial function, although memory may also be affected [5, 7]. However, there is an important cognitive heterogeneity between patients, especially in the rate of cognitive decline [8]. Some of this variability is thought to be related to extrinsic factors and comorbidities, but genetics may also play an important role. A recent systematic review highlighted the role of the genetic risk factors for cognitive decline in PD, with an especial focus on some genetic forms of the disease [9]. Along with pathogenic mutations, genetic variants in at least 3 genes, apolipoprotein *E* (*APOE*), microtubule-associated protein tau (*MAPT*), and *α*-synuclein (*SNCA),* might play a role in determining susceptibility to cognitive impairment in PD [10]. Postmortem studies have revealed that cortical and limbic *α*-synuclein pathology is the hallmark of PD dementia. However, coexistent pathology such as amyloid plaques, tau-related pathology, and vascular lesions may coexist and contribute to cognitive decline in PD [11, 12].

Although most cases of PD are sporadic, up to 10% of PD patients have family history suggesting an important genetic contribution [13]. The genetic basis of PD is complex and includes monogenic forms of PD and genetic risk factors. Autosomal dominant PD is linked to mutations in the *SNCA*, leucine-rich repeat kinase 2 (*LRRK2*), and vacuolar sorting protein 35 (*VPS35*) genes. Common genes causing autosomal recessive PD include *Parkin*, PTEN-induced putative kinase 1 (*PINK1*), and *DJ-1*. More recently, some genetic risk factors for PD have been recognized, such as mutations in the glucocerebrosidase (*GBA*) gene [14].

We aimed to review the literature on cognitive impairment of the most common forms of genetic PD. We focus on clinical studies about cognitive function in genetic PD and summarize findings on neuroimaging and biological biomarkers of cognitive decline in each genetic form of the disease.

## 2. Autosomal Dominant Inheritance

### 2.1. Leucine-Rich Repeat Kinase (*LRRK2*)-Associated PD (*LRRK2*-PD) (PARK8)

Mutations in the *LRRK2* gene are the most common cause of autosomal dominant PD, accounting for approximately 5% of familial and 1% of sporadic PD [15]. However, some populations have a higher incidence, such as 20% in the case of PD patients of Ashkenazi Jewish ancestry [16] and 40% of North African Berber Arab PD patients [17]. Although 132 mutations have been reported in the *LRRK2* gene, only seven have been proven to be pathogenic. These include p.G2019S, p.R1441C/G/H, p.N1437H, p.Y1699C, p.S1761R, p.I2012T, and p.I2020T mutations, being p.G2019S the most frequent worldwide [15, 18]. *LRRK2*-PD is clinically similar to idiopathic PD (IPD), although some differences have been reported, such as less hyposmia, good response to L-DOPA, late age at onset, and absence of atypical signs [15, 19–21].

Cognitive decline has been reported in approximately 23% of *LRRK2*-PD patients in a recent systematic review [21]. Several cross-sectional studies have compared the cognitive profile of *LRRK2*-PD patients with IPD patients (Table 1). In most of them, the tests performed to assess the cognitive state were short screening tests such as the Mini-Mental State Examination (MMSE) and the Montreal Cognitive Assessment (MoCA), finding no differences between *LRRK2*-PD and IPD patients [23–25, 31, 33]. More detailed studies, which included a detailed neuropsychological assessment, have shown inconsistent results. Some have shown a better cognitive performance among *LRRK2*-PD patients compared with IPD. Srivatsal et al. found that *LRRK2*-PD p.G2019S and p.R144G carriers performed better than IPD patients on working memory tests [28]. Somme et al. observed that *LRRK2*-PD p.R1441G carriers had a better performance than IPD in the Mattis Dementia Rating Scale (MDRS) and also in episodic verbal memory tests [29]. Finally, Alcalay and colleagues reported that *LRRK2*-PD p.G2019S carriers performed better than IPD on attention and language tests [30]. The neuropathological findings in *LRRK2*-PD patients could explain, at least partly, the better cognitive profile in *LRRK2*-PD compared with IPD, since a significant proportion of *LRRK2*-PD patients do not show the presence of abnormal aggregates of *α*-synuclein; e.g., Lewy bodies (LB) and clinicopathological correlations have shown that the presence of LB is associated with cognitive impairment and dementia [34, 35]. However, other studies have shown no differences in cognition between *LRRK2*-PD and IPD [22, 26, 27]. In a recent systematic review, the analysis of the data reported in the literature suggested that *LRRK2*-associated disease may have a milder cognitive phenotype than IPD [9]. However, more recent studies do not support this conclusion. A prospective study of a large cohort of PD patients of Ashkenazi Jewish descent, both with and without the p.G2019S *LRRK2* mutation, assessed changes in cognition along time using the MoCA test. Although there was a trend toward better scores among *LRRK2*-PD patients, statistically significant differences were not found [32]. Importantly, the heterogeneity of the patient's cohorts assessed, including several mutations, the different ages, a high heterogeneity in the cognitive tests performed, and some ethnic and cultural aspects, probably influences the wide variety of results obtained.

Cognition was also investigated in asymptomatic *LRRK2* mutation carriers, a population at risk of developing PD. Two studies have compared the cognitive function between healthy relatives of Ashkenazi PD patients carrying the p.G2019S mutation in the *LRRK2* gene and healthy relative noncarriers of the *LRRK2* mutation. While Thaler et al. found a poorer performance on executive function tests among asymptomatic *LRRK2* p.G2019S carriers [36], Mirelman et al. did not observe statistically significant differences among groups [37]. Other studies in asymptomatic *LRRK2* carriers did not show differences in other non-motor symptoms [38]; however, the study of the prodromal phase in *LRRK2*-PD is still ongoing.

To summarize, available data regarding the cognitive profile of *LRRK2*-PD patients include several cross-sectional studies but only one longitudinal study. Overall, these studies have shown a trend toward milder cognitive performance in *LRRK2*-PD compared with IPD. Longitudinal studies with homogenous ethnic group, large sample sizes, and comprehensive neuropsychological battery comparing cognitive outcomes between *LRRK2*-PD and IPD are needed to confirm these findings.

### 2.2. *α*-Synuclein (*SNCA)*-Associated PD (PARKIN1)

Mutations in the *SNCA* gene, which encodes *α*-synuclein protein, were the first discovered genetic cause of familial PD [39]. However, the frequency of *SNCA* mutations as a cause of familial PD is very low, accounting for approximately 2% of autosomal dominant cases [13]. To date, there are 7 missense mutations (p.A30P, p.E46K, p.H50Q, p.G51D, p.A53E, p.A53T, and p.A53V) and gene multiplications (duplications and triplications) reported to cause familiar PD [40, 41]. Also, the p.A18T and p.A29S substitutions were described, but their role as pathogenic has not been probed yet [42]. The clinical phenotype varies according to the mutations. Overall, *SNCA*-PD is associated with an earlier age of disease onset, faster motor progression, early occurrence of motor fluctuations, and prominent non-motor features, compared with IPD [43]. Patients with *SNCA* triplications, compared to those with duplications, have an earlier disease onset, a more rapidly progressive course, and are more often associated with dementia and dysautonomia [44].

The majority of *SNCA*-PD patients described have cognitive impairment and dementia with psychiatric symptoms such as delusions and visual hallucinations [45–50]. Systematic reviews have shown that the prevalence of dementia varies according to the type of SNCA mutation: 39% in p.A53T, 80% in p.H50Q, 50% in p.A30P, 80% in p.E46K, 50–70% of duplication in the *SNCA* carriers, and 88–100% of triplication in the *SNCA* gene reported [21, 43]. In the last systematic review, no differences in the frequency of cognitive decline were observed between the different mutations [21]. The occurrence of cognitive decline varies according to the mutation, but has been described early in the majority of cases, between 2 and 10 years from the onset of the motor symptoms [43]. According to the gene dosage effect, differences in the age at onset of dementia were observed between patients with *SNCA* duplications and triplications, with 57 ± 11 years for duplication carriers and 39 ± 4 years for triplication carriers [40, 43].

Although the profile of the cognitive impairment in *SNCA*-PD is not well characterized, some studies included a neuropsychological assessment, reported language and speech impairment [46, 50], and a decreased performance in visuospatial construction and executive function tasks [51, 52] among patients carrying the p.A53T mutation, which is the most common missense mutation. In addition, a few p.A53T *SNCA* mutation carriers and *SNCA* duplication carriers have been described with a rapid cognitive decline predominantly affecting executive and frontal/subcortical functions [53, 55]. The clinical severity of the disease seems to correlate with the *SNCA* copy number in *SNCA* multiplications, with PD patients who carry triplications having a more severe disease progression and worse cognitive deficit than those with duplications [44].

The neuropathological features of *SNCA*-PD patients are similar to those with IPD, with abnormal aggregates of pathological *α*-synuclein, e.g., LB, in the brainstem and cerebral cortex. Cortical neuronal loss particularly in the hippocampal formation has also been observed and some cases with tau inclusions and TAR DNA-binding protein 43 (TDP-43) pathology [50, 56]. The cortical involvement seen in the autopsies may explain clinical dementia in the majority of patients.

In conclusion, cognitive decline is common among *SNCA* mutation carriers, being those patients with triplications of *SNCA* gene the most affected. However, since *SNCA*-PD is uncommon, data comparing cognitive function between *SNCA*-PD and IPD are scarce, and only a few studies include a complete neuropsychological assessment.

## 3. Autosomal Recessive Inheritance

### 3.1. *Parkin*-Associated PD (PARK2)

*Parkin* gene encodes the parkin protein, a ubiquitin E3 ligase involved in the proteasome degradation pathway [57]. Mutations in the *Parkin* gene are the most common cause of autosomal recessive early-onset PD (EOPD), being present in approximately 15.5% of familial and 4.3% of sporadic cases EOPD cases [58]. The clinical phenotype of *Parkin*-PD is a predominantly early-onset parkinsonism, starting in the third decade of life, with a frequent symmetrical involvement, limb dystonia at onset, slow disease progression, and greater incidence of levodopa-induced dyskinesias compared with IPD [59–61]. Several pathogenic mutations have been reported, including missense and nonsense mutations, but also copy number mutations (deletions and duplications). Mutations have been described in the homozygous, compound heterozygous, and heterozygous states, but the role of heterozygous mutations remains controversial [62, 63].

*Parkin*-PD has previously been defined as cognitively benign, and only a few cross-sectional studies have evaluated the cognitive profile in patients with this genetic form of PD (Table 2). Studies in which the MMSE or MoCA test was applied found a similar cognitive profile between *Parkin*-PD and IPD patients [33, 61, 64]. However, the MMSE is likely not enough sensitive to detect subtle cognitive changes in a younger, nondemented group. Three cross-sectional studies included a neuropsychological evaluation. Lohmann et al. found a similar cognitive performance among *Parkin*-PD and IPD patients [65], results similar to those found by Caccappolo et al. [66]. In contrast, another study with *Parkin*-EOPD patients, with longer disease duration, found that *Parkin*-EOPD performed better in tests of attention, memory, and visuospatial cognitive domains [67]. This relative cognitive preservation might be explained by the neuropathological findings observed in *Parkin*-PD, with neuronal loss in the substantia nigra without LB pathology in the majority of cases, or with LB limited to brainstem areas [56].

In summary, studies suggest that cognitive function in *Parkin*-PD is at least similar or even better than IPD. A more in-depth neurocognitive evaluation in younger patients and a longitudinal follow-up study are required to confirm the suspected slower disease cognitive progression in these patients. There are no data on imaging or biological cognitive biomarkers in *Parkin*-PD.

### 3.2. Phosphatase and Tensin Homolog-Induced Putative Kinase 1 (*PINK1*)-Associated PD

*PINK1* mutations are the second most common cause of autosomal recessive EOPD, accounting for 1–8% of familial PD and less than 1% of sporadic EOPD [68, 69]. The clinical phenotype is similar to P*arkin*-PD, characterized by early-onset parkinsonism, slow disease progression, and good response to levodopa and dystonia [70, 71]. In addition, psychiatric features such as anxiety and depression are common [72].

Since *PINK1*-PD is rare, cognition has never been extensively investigated and there are no data comparing cognitive function between *PINK1*-PD and IPD. Some *PINK1-*PD cases reported in the literature have a mild cognitive impairment [73–75], but in a recent systematic review of genetic autosomal recessive PD patients, cognitive decline was reported in 14% of *PINK1*-PD patients [59], suggesting a low rate of cognitive decline among the patients with this genetic form of PD. The neuropathological data in *PINK1*-PD are very limited. There is only one brain autopsy described in the literature that had LB pathology in the reticular nuclei of the brainstem, substantia nigra, and Meynert nucleus, and absence of tau or TDP-43 inclusions [76].

In conclusion, data regarding cognitive function in *PINK1*-PD is scarce, overall suggesting that cognitive decline is rare in this genetic form of PD. The multicenter longitudinal follow-up studies are needed for a better characterization of cognition in these patients.

## 4. Risk Factors for PD

### 4.1. Glucocerebrosidase (*GBA*)-Associated PD

*GBA* gene encodes the lysosomal enzyme glucocerebrosidase, implicated in the metabolism of glucosylceramide. Pathogenic mutations in both alleles of *GBA* cause the recessive lysosomal storage disorder Gaucher's disease, and heterozygous *GBA* mutations are the most common genetic risk factor for PD and dementia with Lewy bodies (DLB) [77, 78]. A multicenter study identified *GBA* mutations in 3% of PD patients and found that *GBA* mutations increase the risk of PD by approximately fivefold [78].

The clinical phenotype of *GBA*-PD seems to be different from IPD, with an earlier age at onset, significant association with akinetic rigid onset, and more severe non-motor symptoms including cognitive changes [78–81]. The frequency of cognitive decline or dementia is significantly higher in *GBA*-PD compared with IPD (48% vs 24–31%, approximately) [82–85]. Setó-Salvia et al. found that the individual risk of dementia in *GBA*-PD is increased sixfold compared with IPD. Furthermore, in a retrospective review, dementia and psychosis developed significantly earlier in *GBA*-PD compared with IPD [86]. Recently, an Italian study has shown that different types of *GBA* mutations underlie distinct phenotypic profiles, demonstrating that severe and complex *GBA-*PD mutations have a higher risk and earlier occurrence of hallucinations and cognitive impairment compared with mild *GBA* mutations [81]. The neuropathological studies from *GBA*-PD patients revealed a widespread LB pathology, involving both brainstem and cortical areas, which could explain the cognitive impairment in these patients. Moreover, coexistent Alzheimer's disease pathology has also been reported [56, 87].

Several studies have tried to characterize the cognitive profile in *GBA*-PD patients (Table 3). In those studies, in which a cognitive screening test was used to assess the cognitive function in these patients results were conflicting. While most of them found no significant differences among *GBA*-PD and IPD patients [64, 88, 89, 92, 94], others observed a worse performance in *GBA*-PD [79, 82]. Some of these conflicting results could be explained, at least partly, by methodological issues. For example, in the study of Malek et al. patients included were at early stages of the disease (average disease duration 1.5 years) and patients with dementia were excluded [92]. However, other studies include *GBA*-PD patients regardless of their cognitive state. Also, the way to characterize the cognitive profile varies among studies. Only two studies in *GBA*-PD used a complete neuropsychological battery [82, 90]. In both, *GBA*-PD performed worse than noncarriers in different cognitive domains, such as nonverbal memory and visuospatial domains [82], executive function, working memory, and visuospatial domains [90]. Two longitudinal prospective studies with a small sample size showed inconsistent results regarding progression to dementia among *GBA*-PD compared with IPD [89, 91]. It is also possible that the different *GBA* mutations play a different role in the cognitive impairment in *GBA*-PD patients. In line with this hypothesis, Cilia et al. showed that the risk of dementia is modulated by the type of mutation in *GBA* carriers, with a higher risk of dementia in subjects with severe mutations (p.L444P, p.G377S, IVS10+1G > *T*) compared with mild mutations (p.N370S) [95]. In addition, one of the largest PD genome-wide association studies (GWAS) that includes longitudinal data from multiple cohorts showed that GBA variant p.E326K was associated with the rate of cognitive decline (2.37-fold higher odds of having cognitive impairment at baseline and 2.78-fold higher hazard ratio of developing cognitive impairment during follow-up) [96].

Data regarding cognition in asymptomatic *GBA* carriers are scarce. Some cross-sectional studies have found a worse performance on MoCA test among asymptomatic *GBA* carriers compared with controls [88], while others found no significant differences between these two groups of subjects [97, 98]. Regarding longitudinal prospective studies, Mullin et al. found evidence of deteriorating cognition among asymptomatic *GBA* carriers using the MoCA test over 4–5 years [99], while Avenali et al. found no differences over 6 years and suggested that this could be attributable to a training effect as participants repeated the same test multiple times [100].

In conclusion, *GBA*-PD is associated with more severe cognitive impairment, in particular greater impairment in executive and visuospatial domains, and, possibly, a more rapid disease progression. Long-term larger follow-up studies are required to determine the progression over time of cognitive decline in these patients.

## 5. Biological Markers of Cognitive Decline in Genetic PD

Biomarkers that reflect the pathological processes underlying cognitive dysfunction in PD are still under investigation. The neuropathology underlying dementia in IPD is not well established: several studies demonstrate an association between the presence of cortical Lewy pathology and cognitive decline in PD, but multiple comorbid pathologies can occur in patients with PD and cognitive decline, including cerebrovascular disease, argyrophilic grain disease, hippocampal sclerosis, and Alzheimer's disease (AD) pathology. Significant efforts to identify biomarkers that reflect the presence of proteinopathy and neurodegeneration related to cognitive decline in IPD have been made [34]. Alpha-synuclein levels in CSF were demonstrated to be lower in PD compared with controls but do not seem to differentiate between patients with or without dementia [101]. The results from studies investigating CSF levels of total tau or phosphorylated tau as an indicator of cognitive dysfunction in PD have been inconsistent. However, several studies examining beta-amyloid have found that lower CSF levels of beta-amyloid 1–42 (Ab42), the major component of amyloid-b plaques, are associated with worse cognition and that CSF Ab42 levels may predict cognitive decline in PD (in et al, 2015).

In genetic PD, the neuropathological correlates of cognitive decline and the development of biomarkers of cognitive decline are still scarcely investigated. CSF biomarkers of cognitive decline have been rarely assessed in *LRRK2*-PD. *α*-synuclein levels in CSF in *LRRK2*-PD have been recently explored showing higher levels in *LRRK2*-PD compared with IPD, but their correlation with cognitive decline has not been explored [100–102]. Studies measuring CSF levels of amyloid-beta (Ab1-42), total Tau (t-Tau) and phosphorylated Tau (p-Tau) in *LRRK2*-PD, and asymptomatic LRRK2 carriers and IPD patients showed no differences between groups [105, 106] Mov Disord 2016, although their correlation with cognitive decline in this form of genetic PD has been not investigated yet. Since most cases of *LRRK2*-PD have the classic neuropathology of PD but a significant subset lacks Lewy pathology, we could hypothesize that the levels of AD biomarkers are normal in this genetic form of PD. However, interestingly, cognitive impairment and dementia are correlated with the presence of Lewy pathology in *LRRK2*-PD [35].

In contrast to *LRRK2*-PD, *SNCA*-PD and *GBA*-PD can often have prominent cognitive dysfunction and cortical Lewy pathology. There are only a few cases of *SNCA*-PD described in the literature with CSF examination. Two patients with PD and dementia, carriers of a duplication in the *SNCA* gene, showed low levels of *α*-synuclein in the CSF [107]. Seven patients with the mutation p.A53T in the *SNCA* gene (five PD patients and two asymptomatic carriers) had normal t-Tau and p-Tau CSF levels and marginally decreased Ab1-42 levels in 2 out of the 5 symptomatic carriers [53], not related to the cognitive decline. Until now, just a few studies tried to investigate biomarkers of cognitive decline in CSF of *GBA*-PD patients. Although lower levels of Ab1-42 were reported in *GBA*-PD, compared with healthy controls [106], these results were not lately replicated [108]. Recently, it has been suggested that the effects of *GBA* mutations on CSF *α*-synuclein profiles and phenotypical characteristics seem dependent on *GBA* mutation severity, since PD patients carrying severe *GBA* mutations showed more pronounced cognitive decline and reduced CSF levels of total *α*-synuclein in the CSF [109].

## 6. Functional Imaging Markers of Cognitive Decline in Genetic PD

Several brain imaging modalities have been explored to identify measures of brain dysfunction that could serve as biomarkers of cognitive impairment in IPD. Reductions in cortical glucose metabolism were correlated with performance on neuropsychological tests, and reductions in parietal lobe metabolism were associated with the risk of cognitive decline in newly diagnosed PD patients [110]. Other functional imaging approaches such as resting-state functional MRI showed that cognitive decline in PD seems to be associated with disruption of corticostriatal and frontal cortex functional connectivity.

A few studies trying to explore imaging markers of cognitive decline in genetic PD patients were performed. Functional neuroimaging techniques to evaluate cerebral metabolic abnormalities related to cognitive decline have been sparsely investigated in *LRRK2*-PD. De Rosa et al. found a less severe posterior cortical hypometabolism in *LRRK2*-PD compared with IPD, although the cognitive profile was similar between both groups [111]. Abnormalities in functional connectivity were observed in *LRRK2*-PD patients and also in asymptomatic *LRRK2* carriers [103, 112–116], but the relationship between functional connectivity changes and cognitive performance in *LRRK2*-PD is still unclear.

Some patients with *SNCA*-PD and dementia were evaluated by means of single-photon emission computed tomography (SPECT). The patients with *SNCA* duplications showed hypoperfusion of the frontotemporal and occipital lobes [117, 118], whereas a frontoparietal lobe hypoperfusion was observed in patients with p.A53T and p.G51D mutations [49, 51]. However, the significance of these hypoperfusion patterns and its role as a marker of cognitive decline is not known yet. Regarding *GBA*-PD, there is only one study, which assessed cerebral perfusion, by means of SPECT, compared with IPD and DLB [95]. *GBA*-PD and DLB patients had a similar cerebral perfusion pattern, with a significant hypoperfusion in posterior parietal and occipital regions compared with IPD.

Future studies in prospective cohorts of patients, using neuroimaging techniques and CSF biomarkers, are needed to characterize the cognitive decline in genetic PD and to elucidate the role of CSF biomarkers to assess cognitive decline in this specific disease.

## 7. Conclusions

The different forms of genetic PD present variable proportions of cognitive involvement. In autosomal dominant forms of PD, *SNCA*-PD is most frequently associated with cognitive impairment, with certain mutations clearly increasing the risk of early dementia, such as *SNCA* triplications and the p.E46 K mutation. Among *LRRK2*-PD patients, the frequency of cognitive impairment is similar or lower than that observed in IPD. Recessive forms of familial PD, including *Parkin*-PD and *PINK1*-PD, are generally characterized by a lower frequency of cognitive impairment. The risk factors for PD such as *GBA* gene mutations have shown an increased risk of dementia. The variable neuropathological findings in the genetic forms of PD could explain, at least partly, the different cognitive involvements in each form of genetic PD. Also, the coexistence of AD pathology and other neurodegenerative disorders might contribute to the cognitive decline. Future prospective large-scale studies in patients with genetic PD, as well as high-risk susceptibility loci for PD, are needed to better characterize genetic contributions for cognitive decline in PD. The ongoing development in the field of biological and imaging biomarkers of cognitive decline could help to identify those patients with genetic PD in a higher risk of developing a cognitive decline.

## Figures and Tables

**Table 1:** Studies assessing cognition in *LRRK2*-associated PD.

	Participants	Ethnicity	Type of study	Cognitive measures	Findings
Belarbi et al. [22]	23 *LRRK2*-PD (all p.G2019S) 48 IPD	Algerian	Cross-sectional	MMSE, MDRS, FAB, neuropsychological battery	No significant differences
Shanker et al. [23]	21 *LRRK2*-PD (all p.G2019S) 21 IPD	Ashkenazi Jewish	Cross-sectional	MMSE Hopkins verbal learning test Judgment line orientation test FAB	No significant differences
Ben Sassi et al. [24]	55 *LRRK2*-PD (all p.G2019S) 55 IPD Tunisian cohort	Maghrebi	Cross-sectional	MMSE, MoCA, FAB	No significant differences
Mirelman et al. [25]	50 *LRRK2*-PD all p.G2019S) 50 IPD	Ashkenazi Jewish	Cross-sectional	MoCA, trail-making tests A and B, verbal fluency, digit span, and Stroop test	No significant differences
Estanga a et al. [26]	30 *LRRK2*-PD (all p.R1441C) 30 IPD	Caucasian	Cross-sectional	MDS criteria for PD-MCI and PDD Neuropsychological battery	No significant differences
Zheng et al. [27]	45 *LRRK2*-PD (all S1647T) 45 IPD	Asian	Cross-sectional	MMSE Neuropsychological battery	No significant differences
Srivatsal et al. [28]	29 *LRRK2*-PD (24 with p.G2019S and 5 with p.R1441C) 1326 IPD	Caucasian	Cross-sectional	MMSE Clinical diagnosis of dementia Neuropsychological battery	*LRRK2*-PD performed better on MMSE and working memory, and had lower frequency of dementia (4% vs 19.6%)
Somme et al. [29]	27 *LRRK2*-PD (all p.R1441G) 27 IPD	Caucasian	Cross-sectional	Semistructured interview (subjective cognitive complaints) MDRS neuropsychological battery	*LRRK2*-PD performed better on general cognition (MDRS) and episodic verbal memory
Alcalay et al. [30]	116 *LRRK2*-PD (all p.G2019S) 120 IPD	Ashkenazi Jewish	Cross-sectional	Neuropsychological battery	*LRRK2*-PD performed better on attention and language tasks
Hoon et al. [31]	23 *LRRK2*-PD (all G2385R) 276 IPD	Asian	Cross-sectional	MMSE, MoCA (Korean version)	No significant differences
Saunders-Pullman et al. [32]	144 *LRRK2*-PD (all p.G2019S) 401 IPD	Ashkenazi Jewish	Longitudinal	MoCA	No significant differences. A trend toward higher score in MoCA in *LRRK2*-PD
Tan et al. [33]	18 LRRK2-PD (16 with p.G2019S and 2 with p.R1441C) 2082 IPD	Caucasian	Cross-sectional	MoCA	No significant differences

PD = Parkinson's disease; IPD = idiopathic Parkinson's disease; MoCA = Montreal Cognitive Assessment; MMSE = Mini-Mental State Examination; MDRS = Mattis Dementia Rating Scale; USA = United States of America; MDS = Movement Disorders Society; PD-MCI = Parkinson's disease mild cognitive impairment; PDD = Parkinson's disease dementia; FAB = frontal assessment battery.

**Table 2:** Studies assessing cognitive function in *Parkin*-associated PD.

	Participants	Type of study	Cognitive measures	Findings
Luking et al. [61]	101 *Parkin*-EOPD (≤45 years)	Cross-sectional	MMSE	No significant differences
	85 EOPD noncarriers			
Alcalay et al. [64]	43 *Parkin*-EOPD (≤50 years)	Cross-sectional	MMSE	No significant differences
	596 *LRRK2-*EOPD, *GBA*-EOPD, and noncarriers			
Lohmann et al. [65]	21 *Parkin*-EOPD (<45 years)	Cross-sectional	MMSE, MDRS, Grober and Buschke test, WCST, TMT, FAB	No significant differences
	23 EOPD noncarriers			
	9 asymptomatic *Parkin* carriers			
Caccappolo et al. [66]	43 *Parkin*-EOPD (≤50 years)	Cross-sectional	Neuropsychological battery	No significant differences
	52 EOPD noncarriers			
	217 controls (146 noncarriers and 71 asymptomatic *Parkin* carriers)			
	CORE-PD cohort			
Alcalay et al. [67]	21 *Parkin*-EOPD (≤50 years) and long duration disease (>14 years)	Cross-sectional	Neuropsychological battery	*Parkin*-PD performed better on tests of attention, memory, and visuospatial cognitive domains
	23 EOPD noncarriers			
	CORE-PD cohort			
Tan et al. [33]	9 Parkin-EOPD (≤50 years)	Cross-sectional	MoCA	*Parkin-*PD performed better
	202 EOPD noncarriers			

EOPD = early-onset Parkinson's disease; MMSE = Mini-Mental State Examination; MDRS = Mattis Dementia Rating Scale; WCST = Wisconsin card sorting test; TMT = trail-making test; FAB = frontal assessment battery.

**Table 3:** Studies assessing cognitive function in *GBA*-associated PD.

	Participants	Type of study	Cognitive measure	Findings
Alcalay et al. [64]	37 *GBA-EO*PD (≤50 years)	Cross-sectional	Self-reported cognitive impairment	*GBA*-PD reported more self-cognitive impairment compared with *LRRK2-*PD, *Parkin*-PD, and IPD
	596 EOPD (*LRRK2-PD*, *Parkin-PD,* and noncarrier PD)		MMSE	There were no significant differences among the genetic groups in MMSE
Brockmann et al. [79]	20 *GBA*-PD	Cross-sectional	MoCA	*GBA*-PD performed worse than IPD
	20 IPD			
Alcalay et al. [82]	33 *GBA*-PD (≤50 years)	Cross-sectional	MMSE	*GBA*-PD performed worse on the MMSE, memory, and visuospatial domains
	60 EOPD noncarriers of any genetic mutation		Neuropsychological battery	Clinical diagnosis of MCI or dementia more frequent in *GBA*-PD
	CORE-PD cohort		Clinical diagnosis (normal, MCI, dementia)	
Mc Neil et al. [88]	30 Gaucher's disease patients	Cross-sectional	MMSE	Gaucher's disease patients and *GBA*-PD performed worse in MoCA compared with controls; no significant differences in MMSE score
	30 *GBA*-PD		MoCA	
	30 controls			
Chanine et al. [83]	20 *GBA*-PD	Cross-sectional	Consensus clinical determination	*GBA*-PD more likely to have MCI or dementia
	242 IPD			
Setó-Salvia et al. [85]	22 *GBA*-PD	Cross-sectional	Diagnosis of dementia based on a score ≥1 on the CDR and criteria of the MDS IV-TR	Higher prevalence of dementia in *GBA*-PD than in noncarriers
	225 IPD			
Winder-Rhodes et al. [89]	9 *GBA*-PD vs 250 non-*GBA*-PD (cross-sectional phase)	Cross-sectional and longitudinal	MMSE	No significant differences in MMSE comparing *GBA*-PD and noncarriers
	4 *GBA*-PD vs 106 noncarrier PD (longitudinal phase)		Diagnosis of dementia	*GBA* carriers had an increased risk of conversion to dementia (RR 5.45)
Mata et al. [90]	60 *GBA* mutation carrier PD	Cross-sectional	Neuropsychological battery	*GBA-*PD and p.E326 K performed worse in working memory/executive function and visuospatial function; also, more proportion of dementia in both groups
	65 p.E326K polymorphism carriers			
	1055 IPD			
Davis et al. [91]	27 *GBA* mutation carrier PD	Prospective longitudinal	Neuropsychological battery	p.E326 K PD had a higher proportion of progression to MCI and dementia
	32 p.E326K polymorphism carriers		Progression to MCI and dementia	
	674 IPD			
Malek et al. [92]	142 *GBA*-PD	Cross-sectional	MoCA	No significant differences
	1584 IPD			
	Newly diagnosed PD patients (average disease duration 1.5 years)			
Biswas et al. [93]	198 IPD	Cross-sectional	Neuropsychological battery	Impaired recent memory was significantly associated with p.L444P carriers
	136 PD with cognitive impairment			
	184 Parkinson plus 46 Alzheimer disease			
	241 controls			

EOPD = early-onset Parkinson's disease; IPD = idiopathic Parkinson's disease; MMSE = Mini-Mental State Examination; MoCA = Montreal Cognitive Assessment; MCI = mild cognitive impairment; CDR = Clinical Dementia Rating; RR = relative risk.

## References

[1] Lawson R. A., Yarnall A. J., Duncan G. W. (2017). Stability of mild cognitive impairment in newly diagnosed Parkinson’s disease. *Journal of Neurology Neurosurgery and Psychiatry*.

[2] Santangelo G., Vitale C., Picillo M. (2015). Mild cognitive impairment in newly diagnosed Parkinson’s disease: a longitudinal prospective study. *Parkinsonism & Related Disorders*.

[3] Aarsland D., Andersen K., Larsen J. P. (2003). Prevalence and characteristics of dementia in Parkinson disease: an 8 year prospective study. *Archives of Neurology*.

[4] Hely M. A., Reid W. G., Adena M. A. (2008). The Sidney multicenter study of Parkinson’s disease: the inevitability of dementia at 20 years. *Movement Disorders*.

[5] Aarsland D., Bronnick K., Williams-Gray C. (2010). Mild cognitive impairment in Parkinson disease: a multicenter pooled analysis. *Neurology*.

[6] Hughes T. A., Ross H. F., Musa S. (2000). A 10-year study of the incidence of and factors predicting dementia in Parkinson’s disease. *Neurology*.

[7] Kehagia A. A., Barker R. A., Robins T. W. (2010). Neuropsychological and clinical heterogeneity of cognitive impairment and dementia in patients with Parkinson’s disease. *The Lancet Neurology*.

[8] Aarsland D., Andersen K., Larsen J. P. (2004). The rate of cognitive decline in Parkinson’s disease. *Archives of Neurology*.

[9] Fagan E., Pihlstrom L. (2017). Genetic risk factors for cognitive decline in Parkinson’s disease: a review of the literature. *European Journal of Neurology*.

[10] Mata I. F., Leverenz J. B., Weintraub D. (2014). APOE, MAPT, and SNCA genes and cognitive performance in Parkinson disease. *JAMA Neurology*.

[11] Irwin D. J., White M. T., Toledo J. B. (2012). Neuropathologic substrates of Parkinson’s disease dementia. *Annals of Neurology*.

[12] Smith C., Malek N., Grosset K. (2019). Neuropathology of dementia in patients with Parkinson’s disease: a systematic review of autopsy studies. *Journal of Neurology Neurosurgery and Psychiatry*.

[13] Lesage S., Brice A. (2009). Parkinson’s disease: from monogenic forms to genetic susceptibility factors. *Human Molecular Genetics*.

[14] Sidranski E., Lopez G. (2012). The link between the GBA gene and parkinsonism. *The Lancet Neurology*.

[15] Healy D. G., Flachi M., O’Sullivan S. (2008). Phenotype, genotype, and worldwide genetic penetrance of LRRK2-associated Parkinson’s disease: a case control study. *The Lancet Neurology*.

[16] Ozelius L. J., Senthil G., Saunders-Pullman R. (2006). LRRK2 G2019S as a cause of Parkinson’s disease in Ashkenazi Jews. *New England Journal of Medicine*.

[17] Lesage S., Ibanez P., Lohmann E., Pollak P., Tison F. (2005). LRRK2 mutation in French and North African families with Parkinson’s disease. *Annals of Neurology*.

[18] Clark L. N., Wang Y., Karlins E. (2006). Frequency of LRRK2 mutations in early- and late-onset Parkinson’s disease. *Neurology*.

[19] Alcalay R. N., Mirelman A., Sauners-Pullman R. (2013). Parkinson disease phenotype in Ashkenazi Jews with and without LRRK2 G2019S mutations. *Movement Disoders*.

[20] Gaig C., Vilas D., Infante J. (2014). Nonmotor Symptoms in LRRK2 G2019S associated Parkinson’s disease. *PLoS One*.

[21] Trinh J., Zeldenrust F. M., Huang J. (2018). Genotype-phenotype relations for the Parkinson’s disease genes SNCA, LRRK2, VPS35: MDSGene systematic review. *Movement Disorders*.

[22] Belarbi S., Hecham N., Lesage S. (2010). LRRK2 G2019S mutation in Parkinson’s disease: a neuropsychological and neuropsychiatric study in a large Algerian cohort. *Parkinsonism & Related Disorders*.

[23] Shanker V., Groves M., Heiman G. (2011). Mood and cognition in leucine-rich repeat kinase 2 G2019S Parkinson’s disease. *Movement Disorders*.

[24] Ben Sassi S., Nabli F., Hentati E. (2012). Cognitive dysfunction in Tunisian LRRK2 associated Parkinson’s disease. *Parkinsonism & Related Disorders*.

[25] Mirelman A., Heman T., Yasinovsky K. (2013). Fall risk and gait in Parkinson’s diease: the role of the LRRK2 G2019S mutation. *Movement Disorders*.

[26] Estanga A., Rodriguez-Oroz M. C., Ruiz-Martinez J. (2014). Cognitive dysfunction in Parkinson’s disease realated to the R1441G mutation in LRR2K. *Parkinsonism & Related Disorders*.

[27] Zheng Y., Pei Z., Liu Y. (2015). Cognitive impairments in LRRK2-Related Parkinson’s disease: a study in Chinese Individuals. *Behavioural Neurology*.

[28] Srivatsal S., CHolerton B., Leverenz J. B. (2015). Cognitive profile of LRRK2-Related Parkinson’s disease. *Movement Disorders*.

[29] Somme J. H., Molano Salazar A., Gonzalez A. (2015). Cognitive and behavioral symptoms in Parkinson’s disease patients with the G2019S and R1441G mutations of the LRRK2 gene. *Parkinsonism & Related Disorders*.

[30] Alcalay R. N., Mejia-Santana H., Mirelman A. (2015). Neuropsychological performance in LRRK2 G2019S carriers with Parkinson’s disease. *Parkinsonism & Related Disorders*.

[31] Hong J. H., Kim Y. K., Park J. S. (2007). Lack of association between G2385R and cognitive dysfunction in Korean patients with Parkinson’s disease. *Journal of Clinical Neuroscience*.

[32] Saunders-Pullman R., Mirelman A., Alcalay R. N. (2018). Progression in the LRRK2-associated Parkinson disease population. *JAMA Neurology*.

[33] Tan M., Malek N., Lawton M. (2019). Genetic analysis of Mendelian mutations in a large UK population-based Parkinson’s disease study. *Brain*.

[34] Kalia L. (2018). Biomarkers for cognitive dysfunction in Parkinson’s disease. *Parkinsonism and rel disord*.

[35] Kalia L. V., Lang A. E., Hazrati L. N. (2015). Clinical correlations with Lewy body pathology in LRRK2-related Parkinson disease. *JAMA Neurology*.

[36] Thaler A., Mirelman A., Gurevich T. (2012). Lower cognitive performance in healthy G2019S *LRRK2* mutation carriers. *Neurology*.

[37] Mirelman A., Alcalay R. N., Saunders-Pullman R. (2015). Non-motor symptoms in healty Ashkenazi Jewish carriers of the G2019S mutation in the LRRK2 gene. *Movement Disorders*.

[38] Pont-Sunyer C., Tolosa E., Caspell-Garcia C. (2017). The prodromal phase of leucine-rich repeat kinase 2-associated Parkinson disease: clinical and imaging studies. *Movement Disorders*.

[39] Polymeropoulos M. H., Lavedan C., Leroy E. (1997). Mutation in the alpha-synclein gene identified in families with Parkinson’s disease. *Science*.

[40] Rosborough K., Patel N., Kalia L. V. (2017). A-Synuclein and Parkinsonism: updates and future perspectives. *Current Neurology and Neuroscience Reports*.

[41] Yoshino H., Hirano M., Stoessl A. J. (2017). Homozygous alphasynuclein p.A53V in familial Parkinson’s disease. *Neurobiology of Aging*.

[42] Hoffman-Zacharska D., Koziorowski D., Ross O. A. (2013). Novel A18T and pA29S substitutions in *α*-synuclein may be associated with sporadic Parkinson’s disease. *Parkinsonism & Related Disorders*.

[43] Kasten M., Klein C. (2013). The many faces of alpha-synuclein mutations. *Movement Disorders*.

[44] Ibáñez P., Lesage S., Janin S. (2009). Alpha-synuclein gene rearrangements in dominantly inherited parkinsonism: frequency, phenotype, and mechanisms. *Archives of Neurology*.

[45] Farrer M., Kachergus J., Forno L. (2004). Comparison of kindreds with parkinsonism and alpha-synuclein genomic multiplications. *Annals of Neurology*.

[46] Ikeuchi T., Kakita A., Shiga A. (2008). Patients homozygous and heterozygous for *SNCA* duplication in a family with parkinsonism and dementia. *Archives of Neurology*.

[47] Markopoulou K., Dickson D. W., McComb R. (2008). Clinical, neuropathological and genotypic variability in *SNCA* A53T familial Parkinson’s disease. Variability in familial Parkinson’s disease. *Acta Neuropathologica*.

[48] Spira P. J., Sharpe D. M., Halliday G. (2001). Clinical and pathological features of a Parkinsonian syndrome in a family with an Ala53Thr alpha-synuclein mutation. *Annals of Neurology*.

[49] Tokutake T., Ishikawa A., Yoshimura N. (2014). Clinical and neuroimaging features of patient with early-onset Parkinson’s disease with dementia carrying SNCA p.G51D mutation. *Parkinsonism & Related Disorders*.

[50] Zarranz J. J., Alegre J., Gómez-Esteban J. C. (2004). The new mutation, E46K, of alpha-synuclein causes Parkinson and Lewy body dementia. *Annals of Neurology*.

[51] Puschmann A., Ross O. A., Vilariño-Güell C. (2009). A Swedish family with de novo alpha-synuclein A53T mutation: evidence for early cortical dysfunction. *Parkinsonism & Related Disorders*.

[52] Breza M., Koutsis G., Karadima G. (2018). The different faces of the p.A53T alpha-synuclein mutation: a screening of Greek patients with parkinsonism and/or dementia. *Neuroscience Letters*.

[53] Bougea A., Koros C., Stamelou M. (2017). Frontotemporal dementia as the presenting phenotype of p.A53T mutation carriers in the alpha-synuclein gene. *Parkinsonism & Related Disorders*.

[54] Kara E., Kiely A. P., Proukakis C. (2014). A 6.4 Mb duplication of the *α*-synuclein locus causing frontotemporal dementia and Parkinsonism: phenotype-genotype correlations. *JAMA Neurology*.

[55] Kielb S., Kisanuki Y. Y., Dawson E. (2021). Neuropsychological profile associated with an alpha-synuclein gene (SNCA) duplication. *The Clinical Neuropsychologist*.

[56] Schneider S. A., Alcalay R. N. (2017). Neuropathology of genetic synucleinopathies with parkinsonism: review of the literature. *Movement Disorders*.

[57] Tanaka K., Suzuki T., Chiba T. (2001). Parkin is linked to the ubiquitin pathway. *Journal of Molecular Medicine*.

[58] Kilarski L. L., Pearson J. P., Newsway V. (2012). Systematic review and UK-based study of PARK2 (parkin), PINK1, PARK7 (DJ-1) and LRRK2 in early-onset Parkinson’s disease. *Movement Disorders*.

[59] Kasten M., Hartmann C., Hampf J. (2018). Genotype-phenotype relations for the Parkinson’s disease genes parkin, PINK1, DJ1: MDSGene systematic review. *Movement Disorders*.

[60] Lohmann E., Periquet M., Bonifati V. (2003). How much phenotypic variation can be atributted to parkin genotype?. *Annals of Neurology*.

[61] Lücking C. B., Dürr A., Bonifati V. (2000). Association between early-onset Parkinson’s disease and mutations in the parkin gene. *New England Journal of Medicine*.

[62] Kay D. M., Moran d, Moses L. (2007). Heterozygous parkin point mutations are as common in control subjects as in Parkinson’s patients. *Annals of Neurology*.

[63] Pankratz N., Kissell D. K., Pauciulo M. W. (2009). Parkinson Study Group-PROGENI Investigators. Parkin dosage mutations have greater pathogenicity in familial PD than simple sequence mutations. *Neurology*.

[64] Alcalay R. N., Mejia-Santana H., Tang M. X. (2010). Self-report of cognitive impairment and mini-mental state examination performance in PRKN, LRRK2, and GBA carriers with early onset Parkinson’s disease. *Journal of Clinical and Experimental Neuropsychology*.

[65] Lohmann E., Thobois S., Lesage S. (2009). A multidisciplinary study of patients with early-onset PD with and without parkin mutations. *Neurology*.

[66] Caccappolo E., Alcalay R. N., Mejia-Santana H. (2011). Neuropsychological profile of parquin mutation carriers with and without Parkinson disease: the CORE-PD study. *Journal of the International Neuropsychological Society*.

[67] Alcalay R. N., Caccapolo E., Mejia-Santana H. (2014). Cognitive and motor function in long-duration PARKIN-associated Parkinson disease. *JAMA Neurology*.

[68] Bonifati V. (2012). Autosomal recessive parkinsonism. *Parkinsonism & Related Disorders*.

[69] Kumazawa R., Tomivana H., Li Y. (2008). Mutation analysis of the PINK1 gene in 391 patients with Parkinson disease. *Archives of Neurology*.

[70] Bonifati V., Rohé C. F., Breedveld G. J. (2005). Italian Parkinson Genetics Network. Early-onset parkinsonism associated with PINK1 mutations: frequency, genotypes, and phenotypes. *Neurology*.

[71] Ibáñez P., Lesage S., Lohmann E. (2006). French Parkinson’s disease genetics study group. Mutational analysis of the PINK1 gene in early-onset parkinsonism in europe and North Africa. *Brain*.

[72] Ephraty L., Porat O., Israeli D. (2007). Neuropsychiatric and cognitive features in autosomal-recessive early parkinsonism due to PINK1 mutations. *Movement Disorders*.

[73] Bentivoglio A. R., Cortelli P., Valente E. M. (2001). Phenotypic characterisation of autosomal recessive PARK6-linked parkinsonism in three unrelated Italian families. *Movement Disorders*.

[74] Ibáñez P., Lesage S., Lohmann E. (2006). Mutational analysis of the PINK1 gene in early-onset parkinsonism in Europe and North Africa. *Brain*.

[75] Valente E. M., Salvi S., Ialongo T. (2004). PINK1 mutations are associated with sporadic early-onset parkinsonism. *Annals of Neurology*.

[76] Samaranch L., Lorenzo-Betancor O., Arbelo J. M. (2010). PINK1-linked parkinsonism is associated with Lewy body pathology. *Brain*.

[77] Shiner T., Mirelman A., Gana Weisz M. (2016). High frequency of GBA gene mutations in dementia with lewy bodies among Ashkenazi jews. *JAMA Neurology*.

[78] Sidransky E., Nalls M. A., Aasly J. O. (2009). Multicenter analysis of glucocerebrosidase mutations in Parkinson’s disease. *New England Journal of Medicine*.

[79] Brockmann K., Srulijes K., Hauser A. K. (2011). GBA-associated PD presents with nonmotor characteristics. *Neurology*.

[80] Gan-Or Z., Gilandi N., Rozovski U. (2008). Genotyope-phenotype correlations between GBA mutations and Parkinson disease risk and onset. *Neurology*.

[81] Petrucci S., Ginevrino M., Trezzi I. (2020). Dissection of genotype-phenotype correlates in a large Italian cohort. *Movement Disorders*.

[82] Alcalay R. N., Caccappolo E., Mejia-Santana H. (2012). Cognitive performance of GBA mutation carriers with early-onset PD: the CORE-PD study. *Neurology*.

[83] Chanine L. M., Qiang J., Ashbridge E. (2013). Clinical and biochemical differences in patients having Parkinson’s disease with vs without GBA mutations. *JAMA Neurology*.

[84] Neumann J., Bras J., Deas E. (2009). Glucocerebrosidase mutations in clinical and pathologically proven Parkinson’s disease. *Brain*.

[85] Setó-Salvia N., Pagonabarraga J., Houlden H. (2012). Glucocerebrosidase mutations confer a greater risk of dementia during Parkion’s disease coure. *Movement Disorders*.

[86] Oeda T., Umemura A., Mori Y. (2015). Impact of glucocerebrosidase mutations on motor and nonmotor complications in Parkinson’s disease. *Neurobiology of Aging*.

[87] Clark L. N., Kartsaklis L. A., Wolf Gilbert R. (2009). Association of glucocerebrosidase mutations with dementia with lewy bodies. *Archives of Neurology*.

[88] Mc Neil A., Duran R., Proukakis C. (2012). Hyposmia and cognitive impairment in Gaucher disease patients and carriers. *Movement Disorders*.

[89] Winder-Rhodes S. E., Evans J. R., Ban M. (2013). Glucocerebrosidase mutations influence the natural history of Parkinson’s disease in a community-based incident cohort. *Brain*.

[90] Mata I. F., Leverenz J. B., Weintraub D. (2016). GBA variants are associated with a distinct pattern of cognitive deficits in Parkinson’s disease. *Movement Disorders*.

[91] Davis M. Y., Johnson C. O., Leverenz J. B. (2016). Association of GBA mutations and the E326K polymorphism with motor and cognitive progression in Parkinson’s disease. *JAMA Neurology*.

[92] Malek N., Weil R., Bresner C. (2018). Features of GBA-associated Parkinson’s disease at presentation in the UK Tracking Parkinson’s study. *Journal of Neurology Neurosurgery and Psychiatry*.

[93] Biswas A., Sadhukhan T., Biswas A. (2021). Identification of GBA mutations among neurodegenerative disease patients from eastern India. *Neuroscience Letters*.

[94] Nichols W. C., Pankratz N., Marek D. K. (2009). Mutations in GBA are associated with familial Parkinson disease susceptibility and age at onset. *Neurology*.

[95] Cilia R., Tunesi S., Marotta G. (2016). Survival and dementia in GBA-associated Parkinson’s disease: the mutation matters. *Annals of Neurology*.

[96] Iwaki H., Blauwendraat C., Leonard H. L. (2019). Genetic risk of Parkinson disease and progression: an analysis of 13 longitudinal cohorts. *Neurol Genet*.

[97] Bregman N., Thaler A., Mirelman A. (2017). A cognitive fMRI study in non-manifesting LRRK2 and GBA carriers. *Brain Structure and Function*.

[98] Chanine L. M., Urbe L., Caspell-Garcia C. (2018). Cognition among individuals along a spectrum of increased risk for Parkinson’s disease. *PLoS One*.

[99] Mullin S., Beavan M., Bestwick J. (2019). Evolution and clustering of prodromal parkinsonian features in GBA1 carriers. *Movement Disorders*.

[100] Avenali M., Toffoli M., Mullin S. (2019). Evolution of prodromal parkinsonian features in a cohort of GBA mutation-positive individuals: a 6-year longitudinal study. *Journal of Neurology Neurosurgery and Psychiatry*.

[101] Gao L., Tang H., Nie K. (2015). Cerebrospinal fluid alpha-synuclein as a biomarker for Parkinson’s disease diagnosis: a systematic review and meta-analysis. *International Journal of Neuroscience*.

[102] Aasly J. O., Johansen K. K., Brønstad G. (2014). Elevated levels of cerebrospinal fluid *α*-synuclein oligomers in healthy asymptomatic LRRK2 mutation carriers. *Frontiers in Aging Neuroscience*.

[103] Vilas D., Segura B., Baggio H. C. (2016). Nigral and striatal connectivity alterations in asymptomatic LRRK2 mutation carriers: a magnetic resonance imaging study. *Movement Disorders*.

[104] Garrido A., Fairfoul G., Tolosa E. S., Martí M. J., Green A. (2019). *α*-synuclein RT-QuIC in cerebrospinal fluid of LRRK2-linked Parkinson’s disease. *Annals of Clinical and Translational Neurology*.

[105] Vilas D., Shaw L. M., Taylor P. (2016). Cerebrospinal fluid biomarkers and clinical features in leucine-rich repeat kinase 2 (LRRK2) mutation carriers. *Movement Disorders*.

[106] Brockmann K., Schulte C., Deuschle C. (2015). Neurodegenerative CSF markers in genetic and sporadic PD: classification and prediction in a longitudinal study. *Parkinsonism & Related Disorders*.

[107] Kasuga K., Tokutake T., Ishikawa A. (2010). Differential levels of *α*-synuclein, *β*-amyloid42 and tau in CSF between patients with dementia with Lewy bodies and Alzheimer’s disease. *Journal of Neurology Neurosurgery and Psychiatry*.

[108] Lerche S., Schulte C., Srulijes K. (2017). Cognitive impairment in Glucocerebrosidase (GBA)-associated PD: not primarily associated with cerebrospinal fluid Abeta and Tau profiles. *Movement Disorders*.

[109] Lerche S., Wurster I., Roeben B. (2020). Parkinson’s disease: glucocerebrosidase 1 mutaation severity is associated with CSF alpha-synuclein profiles. *Movement Disorders*.

[110] Firbank M. J., Yarnall A. J., Lawson R. A. (2017). Cerebral glucose metabolism and cognition in newly diagnosed Parkinson’s disease: ICICLE-PD study. *Journal of Neurology Neurosurgery and Psychiatry*.

[111] De Rosa A., Peluso S., De Lucia N. (2018). Cognitive profile and 18F-fluorodeoxyglucose PET study in LRRK2-related Parkinson’s disease. *Parkinsonism & Related Disorders*.

[112] Helmich R. C., Thaler A., van Nuenen B. F. (2015). Reorganization of corticostriatal circuits in healthy G2019S LRRK2 carriers. *Neurology*.

[113] Hou Y., Luo C., Yang J. (2018). Altered intrinsic brain functional connectivity in drug-naïve Parkinson’s disease patients with LRRK2 mutations. *Neuroscience Letters*.

[114] Thaler A., Helmich R. C., Or-Borichev A. (2016). Intact working memory in nonmanifesting LRRK2 carriers–an fMRI study. *European Journal of Neuroscience*.

[115] Thaler A., Mirelman A., Helmich R. C. (2013). Neural correlates of executive functions in healthy G2019S LRRK2 mutation carriers. *Cortex*.

[116] Van Nuenen B. F., Helmich R. C., Ferraye M. (2012). Cerebral pathological and compensatory mechanisms in the premotor phase of leucine-rich repeat kinase 2 parkinsonism. *Brain*.

[117] Nishioka K., Hayashi S., Farrer M. (2006). Clinical heterogeneity of a alpha-synuclein gene duplication in Parkinson’s disease. *Annals of Neurology*.

[118] Nishioka K., Ross O. A., Ishii K. (2009). Expanding the clinical phenotype of *SNCA* duplication carriers. *Movement Disorders*.

